# Relationships between Unilateral Muscle Strength Qualities and Change of Direction in Adolescent Team-Sport Athletes

**DOI:** 10.3390/sports6030083

**Published:** 2018-08-20

**Authors:** Christopher Thomas, Thomas Dos’Santos, Paul Comfort, Paul A. Jones

**Affiliations:** Directorate of Sport, Exercise and Physiotherapy, University of Salford, Salford, Greater Manchester M6 6PU, UK; t.dossantos@edu.salford.ac.uk (T.D.); p.comfort@salford.ac.uk (P.C.); p.a.jones@salford.ac.uk (P.A.J.)

**Keywords:** eccentric, isometric, concentric, countermovement jump, single-leg hop, deceleration

## Abstract

Previous studies have reported an association between global measures of bilateral strength and change of direction (COD) ability. Yet, little is known about the association between unilateral muscle strength qualities and COD ability. The aim of this study was to explore the associations between unilateral muscle strength qualities and COD measures (COD speed (CODS) and COD deficit) when matched limb-for-limb (i.e., right limb vs. right limb, left limb vs. left limb) in adolescent team-sport athletes. One hundred and fifteen athletes (56 males, 59 females) active in cricket, netball, and basketball participated in this investigation. Each player performed trials of countermovement jump (CMJ), single-leg hop (SLH), isometric mid-thigh pull (IMTP) and eccentric knee extensor torque (ECC-EXT) to assess muscle strength qualities and 505 and modified 505 (505_mod_) to evaluate COD ability. Moderate-to-large correlations were observed between SLH and CODS (r = −0.43 to −0.67). Another important finding was that CMJ measures demonstrated moderate-to-large correlations with CODS (r = −0.38 to −0.69) and small-to-moderate correlations with COD deficit (r = −0.24 to −0.45). COD is underpinned by distinct muscle strength qualities and each contribute to specific phases of a COD task. It is therefore likely that such connections exist between muscle strength qualities and COD, with all qualities contributing to overall COD ability.

## 1. Introduction

Change of direction (COD) is a dominant feature of field- and court-based sport athletes and is defined as “the ability to decelerate, reverse or change movement direction and accelerate again, and is considered pre-planned” [1]. Time-motion analysis data of soccer show players performing the equivalent of 727 ± 203 turns during play [2], with players performing 45–49 turns of 90–180° across all positions. Furthermore, elite soccer athletes perform up to 32% of directional changes of 180° [3,4]. At the junior elite level, netball players walking with neutral acceleration and performing a 180° COD contributed 1.5% more to the wider dataset in comparison to the elite standard [5]. Thus, the ability to change direction is important and may strongly influence periods of play whereby athletes are making a break from an opponent, or applying pressure to achieve a turnover [6]. However, despite the increasing research into COD ability from a performance perspective, our understanding of unilateral muscle strength qualities that underpin COD ability remains a major challenge. A deeper understanding of the relationship between unilateral muscle strength qualities and COD ability would provide practitioners with more tangible information on the key physical determinants of COD ability and allow the development of more targeted training strategies to improve performance.

Despite change of direction (COD) ability demonstrating strong associations with concentric [7,8], eccentric [1,7,8,9], isometric [7,8,10], and dynamic strength [7,8], power [11], and reactive strength [12,13], much uncertainty still exists about the relationship between unilateral muscle strength qualities and COD ability. Meylan et al. [14] found unilateral countermovement jump (CMJ) height to demonstrate small associations with COD speed (CODS) in males (r = −0.25) yet moderate associations in females (r = −0.49) when turning on the dominant limb, while moderate-to-large associations were found when turning on the non-dominant limb (r = −0.41 and −0.52, respectively). Castillo-Rodriguez et al. [12] found that unilateral CMJ height of the right limb demonstrated moderate associations (r = −0.47) with modified 505 (505_mod_) CODS, while unilateral CMJ height on the left limb demonstrated small associations (r = −0.21) with 505_mod_ CODS. These findings may suggest CODS of soccer players was associated mainly with CMJ height of the right limb. Yanci et al. [15] found unilateral CMJ height to demonstrate moderate associations to 505 CODS on dominant and non-dominant limbs (r = −0.38 and −0.45, respectively). Similarly, single-leg hop (SLH) has been shown to demonstrate moderate-to-large associations (r = −0.37 to −0.54) with 505 CODS [16]. These results are likely due to the primary application of force throughout the tests being horizontal and lateral [17]. Therefore, it would seem pertinent that horizontal jump assessment would demonstrate superior associations to those activities that involve horizontal linear motion. For instance, Yanci et al. [15] found SLH distance to demonstrate moderate associations to 505 CODS on dominant and non-dominant limbs (r = −0.44 and −0.48, respectively), which were slightly greater than those observed from vertical jump assessments (r = −0.38 to −0.45). Similarly, Meylan et al. [14] found moderate-to-large associations between unilateral horizontal jump distance and CODS on the dominant (r = −0.47 to −0.49) and non-dominant limbs (r = −0.46 to −0.59), respectively.

Given COD ability should be determined by assessing performance of both limbs, its association to muscle strength qualities would be improved by measuring the unilateral muscle strength qualities of both limbs (right and left). There are few studies that have assessed muscle strength qualities using unilateral tests and retained performances of both limbs to determine their association with COD ability (also of both limbs). Therefore, little is known about the relationships between unilateral muscle strength qualities and COD ability, particularly when matched limb-for-limb. With this in mind, the purpose of this investigation was to explore the associations between unilateral muscle strength qualities and COD measures when matched limb-for-limb (i.e., right limb vs. right limb, left limb vs. left limb) in adolescent team-sport athletes.

## 2. Materials and Methods

This investigation included 115 male (*n* = 56) and female (*n* = 59) team-sport athletes. The male athletes participated in basketball (*n* = 17; age = 17.3 ± 0.6 years; height = 187.1 ± 9.4 cm; body mass = 81.6 ± 10.5 kg), cricket (*n* = 23; age = 18.7 ± 2.7 years; height = 175.8 ± 6.1 cm; body mass = 76.9 ± 13.3 kg) and soccer (*n* = 16; age = 20.1 ± 0.6 years; height = 179.1 ± 5.2 cm; body mass = 76.0 ± 8.6 kg), whereas the female athletes participated in netball (*n* = 21; age = 18.1 ± 1.1 years; height = 174.0 ± 6.1 cm; body mass = 66.7 ± 5.1 kg), cricket (*n* = 23; age = 17.6 ± 1.6 years; height = 165.2 ± 9.2 cm; body mass = 61.5 ± 11.1 kg) and soccer (*n* = 15; age = 20.6 ± 0.6 years; height = 168.0 ± 7.2 cm; body mass = 56.2 ± 6.3 kg). Each athlete was in the preseason phase of training during his or her participation in this investigation. All subjects read and signed a written informed consent form before participation, with consent from the parent or guardian of all players under the age of 18. Approval for the investigation was provided by the University of Salford Ethics committee (ethics approval code: HSCR14/129).

A cross sectional observational design was conducted to investigate the relationships between strength (eccentric and isometric) and power (vertical and horizontal jump) measures and CODS (505 and 505_mod_ CODS and 505 and 505_mod_ COD deficit). Maximal isokinetic and isometric strength were selected as both have previously demonstrated strong associations to COD in various populations [1,18], while vertical and horizontal jumps were selected as these are commonly used to assess jump performance [16,19,20,21], and are also largely associated with CODS. Furthermore, 505 and 505_mod_ tests were selected as they are commonly used to assess COD ability (CODS and COD deficit) [7,19].

Unilateral CMJ were performed with one foot on the force platform, and the other limb unsupported and flexed 90° at the knee. Subjects performed CMJs with the hands on the hips, and countermovement depth was self-selected by the subjects to maximize CMJ height and ecological validity with the instruction to “jump as high and as fast as possible”. Each subject performed three warm-up trials on each limb, separated by one minute of rest. Thereafter, subjects performed three maximum effort trials on each limb, with one minute of rest between trials. Countermovement jump data were collected using a portable force platform sampling at 1000 Hz (Kistler Instrument Corporation, Winterthur, Switzerland, Model 9286AA, SN 1209740). The force platform was interfaced with a laptop to allow for direct measurement of force-time characteristics, and then analysed using Bioware software (Version 5.11, Kistler Instrument Corporation, Winterthur, Switzerland) and applied to a customised Microsoft Excel spreadsheet (version 2016, Microsoft Corp., Redmond, WA, USA). Prior to the onset of the countermovement, subjects remained stationary on the force platform for two seconds to enable an accurate measurement of body weight. Vertical ground reaction force data were then averaged across the first second, and the onset of the countermovement was determined 30 ms prior to the instant this value was reduced by 5 standard deviation (SD) [22]. Countermovement jump time to take-off (CMJ-TTT) was calculated from the force-time record as the length of time between the onset of the countermovement and the point of take-off. Jump height (CMJ height) was calculated based on the vertical displacement of the centre of mass based on the laws of constant acceleration. Reactive strength index-modified (CMJ-RSImod) was calculated by dividing CMJ height by CMJ-TTT. The mean performance from each of the 3 trials was used for further analysis.

The SLH was used as a measure of unilateral horizontal jump performance. A 6 m long, 15 cm wide line was marked on the floor, along the middle of which was a standard tape measure, perpendicular to the starting line. The test began with subjects placing the toes on the back of the start line, before balancing on the limb to be tested, with the hands on the hips. Subjects were instructed to use a countermovement, and no restrictions were placed on body angles attained during the preparatory phase, with the instruction to hop as far forward as possible, taking off from one limb, before landing on the same limb. Subjects had to “stick” the landing for two seconds, with no movement of the foot or hands touching the ground, for the trial to be counted. If the subject did not do this, the trial was discarded, and another was attempted. The distance was measured to the nearest 0.01 m using a standard tape measure, perpendicular from the front of the start line to the posterior aspect of the back heel at the landing. Subjects performed a minimum of three warm-up trials on each limb [23], followed by three hops for maximal horizontal distance. The order of limb was randomised and counterbalanced between subjects. The mean hop distance for the three trials was used for further analysis.

Isometric strength was assessed during unilateral stance isometric mid-thigh pull (IMTP) testing, using a portable force platform sampling at 600 Hz (400 Series Performance Force Plate; Fitness Technology, Adelaide, Australia). The force platform was interfaced with computer software (BMS) that allows for direct measurement of force-time characteristics and then analysed using the BMS software (Version 1, Fitness Technology, Adelaide, Australia) and data were filtered using a fourth-order Butterworth filter (Fitness Technology, Adelaide, Australia) with a 16 Hz cut-off frequency. Subjects obtained self-selected knee and hip angles (knee = 130–150°; hip = 140–160°) with one foot in the centre of the force platform, with the other limb unsupported and flexed 90° at the knee. For this test, an immovable, collarless steel bar was positioned at approximately mid-thigh, just below the crease of the hip, using a portable IMTP rig (Fitness Technology, Adelaide, Australia). The bar height could be adjusted (3 cm increments) at various heights above the force platform to accommodate different sized subjects. Once the bar height was established, the subjects stood with feet hip to shoulder-width apart, and their hands were strapped to the bar in accordance with previously established methods [24]. Each athlete was provided two warm-up pulls, one at 50% and one at 75% of the subjects perceived maximum effort, separated by one minute of rest. Once body position was stabilized (verified by watching the subject and force trace), the subject was given a countdown of “3, 2, 1, Pull”. Minimal pretension was allowed to ensure that there was no slack in the subject’s body before initiation of the pull [25]. Subjects were instructed to maintain balance, pull against the bar with maximal effort as quickly as possible, and push their single foot into the force platform. Subjects performed a total of six unilateral maximum effort trials (3 with left and right limbs each), in a randomized order, interspersed with two minutes of recovery between trials. Any trials whereby subjects lost balance were excluded, and further trials were performed after a further 2-min rest period. Prior to the onset of the pull, subjects remained stationary on the force platform for two seconds to enable an accurate measurement of body weight. The onset of movement (defined at time point 0 ms) was the point when force exceeded 40 N from body weight [26]. The maximum force recorded from the force-time curve during the 5-s IMTP trial was reported as the IMTP peak force (IMTP-PF) and was presented as a value relative to body mass (N·kg^−1^). The mean performance from each of the 3 trials for each limb was used for further analysis.

Eccentric knee extensor muscle torque (ECC-EXT) was assessed at 60°·s^−1^ using a Kin Com (Chattanooga Group, Hixson, TN, USA) isokinetic dynamometer, as described in previous research [27]. The dynamometer was calibrated according to manufacturers’ standardised procedures prior to data collection. Before measuring each limb, the motor axis of the dynamometer was visually aligned with the axis of the knee joint (midway between the lateral condyles of the femur and tibia). The cuff of the dynamometer lever arm was attached to the ankle, 5 cm proximal to the malleoli, and the moment arm recorded for gravity correction purposes. The athlete was seated and stabilised by straps so that only the knee to be tested was moving with a single degree of freedom. The back of the seat was adjusted so that the hip angle was 90°. Peak torque was obtained from four maximal repetitions in each mode throughout an arc of 90° (full knee extension = 0°). The resistance provided by the weight of the lower-limb was recorded at 30° knee extension for gravity correction purposes [28], by adding the gravity correction factor: (weight of limb) × (moment arm) × (cosine (angle of flexion)). The highest peak torque of the four repetitions in each mode was used for further analysis and was presented as a value relative to body mass (Nm·kg^−1^). Data were exported in ASCII format into Microsoft Excel (version 2016, Microsoft Corp., Redmond, WA, USA) for further analysis.

Change of direction speed was assessed utilising 505, followed by 505_mod_ tests on a third-generation artificial rubber crumb surface (Mondo, SportsFlex, 10 mm; Mondo America Inc., Mondo, Summit, NJ, USA) using “Brower photocell timing Gates” (model number BRO001; Brower, Draper, UT, USA). Subjects started 0.5 m behind the photocell gates, to prevent any early triggering of the initial start gate, from a two-point staggered start. For the 505, subjects were instructed to sprint to a line marked 15 m from the start line, placing either left or right foot on the line, depending on the trial, turn 180° and sprint back 5 m through the finish. For 505_mod_ testing, subjects were instructed to sprint to a line marked 5 m from the start line, placing either left or right foot on the line, depending on the trial, turn 180° and sprint back 5 m through the finish. During both tests, if the subject changed direction before hitting the turning line, or turned off the incorrect foot, the trial was disregarded, and the subject completed another trial after the rest period. For both tests, subjects performed 3 trials on each limb, in a randomized order, with a 2-min rest between trials. The mean performance from each of the 3 trials, for both 505 and 505_mod_ was used for further analysis.

The COD deficit for both limbs was calculated as the difference between mean 505 or 505_mod_ time and mean 10-m sprint time [29]. Sprint testing was performed on the same surface as the COD trials with timing gates placed at 0-, 5-, 10-, and 20-m. Subjects performed three trials and the mean 10 m sprint time of was used for further analysis. Subsequently, the mean COD deficit from each of the 3 trials for both 505 and 505_mod_ was used for further analysis.

## 3. Statistical Analysis

Data are presented as mean ± SD. Normality of data was assessed by Shapiro-Wilk statistic. Pearson’s product moment correlation or nonparametric equivalent Spearman’s rho (*ρ*) were performed to explore relationships among muscle strength quality to COD ability (CODS and COD deficit) based on pooled, male and female data. Furthermore, all performance measures were categorised as the following: Right limb, left limb, and both limbs averaged. Correlations were evaluated as follows: Small (0.1), moderate (0.3), and large (0.5) [30]. To avoid Type 1 error, a Holm-Bonferroni sequential adjustment was applied as multiple separate comparisons were completed [31]. All statistical analyses were completed using SPSS version 23 (IBM, New York, NY, USA), and statistical significance was set at *p* ≤ 0.05.

## 4. Results

Descriptive data for unilateral muscle strength qualities and COD ability are presented in Table 1. Correlation coefficients between unilateral muscle strength qualities and COD ability are presented in Table 2, Table 3 and Table 4. Correlation coefficients between unilateral muscle strength qualities and COD ability by sport are presented in Tables S1–S3.

### 4.1. Relationships between Muscle Strength Qualities and COD Ability on the Right Limb

- Significant moderate-to-large correlations were revealed between CMJ-RSImod (r = −0.39 to −0.50; *p* < 0.001) and CMJ height (r = −0.38 to −0.60; *p* < 0.001) and CODS (Table 2).
- Significant moderate-to-large correlations were observed between SLH and CODS (r = −0.48 to −0.64; *p* < 0.001). In addition, significant moderate-to-large correlations were revealed between IMTP-PF and CODS (r = −0.39 to −0.51; *p* < 0.001).
- Also, small-to-moderate correlations were found between CMJ-RSImod (R = −0.24 to −0.39; *p* < 0.05), CMJ height (r = −0.30; *p* < 0.05), SLH (r = −0.27 to −0.34; *p* < 0.05), and IMTP-PF (r = −0.39 to −0.40; *p* < 0.05) and COD deficit.

### 4.2. Relationships between Muscle Strength Qualities and COD Ability on the Left Limb

- Significant moderate-to-large correlations were revealed between CMJ-RSImod (r = −0.42 to −0.57; *p* < 0.05) and CMJ height (r = −0.42 to −0.69; *p* < 0.05) and CODS (Table 3).
- Significant moderate-to-large correlations were observed between SLH and CODS (r = −0.43 to −0.65; *p* < 0.05). In addition, significant moderate-to-large correlations were revealed between IMTP-PF and CODS (r = −0.40 to −0.57; *p* < 0.001). Significant moderate correlations were found between ECC-EXT and CODS (r = −0.38 to −0.44; *p* < 0.05).
- Also, small-to-moderate correlations were found between CMJ-RSImod (r = −0.30 to −0.45; *p* < 0.05), CMJ height (r = −0.30 to −0.45; *p* < 0.05), SLH (r = −0.31; *p* < 0.05), and IMTP-PF (r = −0.26 to −0.44; *p* < 0.05) and COD deficit.

### 4.3. Relationships between Muscle Strength Qualities and COD Ability Average across Both Limbs

- Significant moderate-to-large correlations were revealed between CMJ-RSImod (r = −0.40 to −0.57; *p* < 0.05) and CMJ height (r = −0.43 to −0.67; *p* < 0.05) and CODS (Table 4).
- Significant moderate-to-large correlations were observed between SLH and CODS (r = −0.51 to −0.67; *p* < 0.001). In addition, significant moderate-to-large correlations were revealed between IMTP-PF and CODS (r = −0.43 to −0.57; *p* < 0.001). Significant moderate correlations were found between ECC-EXT and CODS (r = −0.39 to −0.49; *p* < 0.05).
- Also, small-to-moderate correlations were found between CMJ-RSImod (r = −0.31 to −0.44; *p* < 0.05), CMJ height (r = −0.34; *p* < 0.001), SLH (r = −0.33; *p* < 0.001), and IMTP-PF (r = −0.34 to −0.40; *p* < 0.05) and COD deficit.

## 5. Discussion

The purpose of this investigation was to explore the associations between unilateral muscle strength qualities and COD measures when matched limb-for-limb (i.e., right limb vs. right limb, left limb vs. left limb) in adolescent team-sport athletes. The current investigation found that SLH was largely associated with COD ability, irrespective of limb or sub-group. Another important finding was that jump performances (CMJ-RSImod and CMJ height) were largely associated with CODS, while measures of lower-limb strength (IMTP-PF and ECC-EXT) were associated with CODS for pooled data and male players. Also, it appears CMJ-RSImod to associate with COD deficit. The present findings suggest that a linear relationship does not always exist when examining associations between muscle strength qualities and COD measures on the same limb. These findings may help us to understand the bilateral nature of COD, specifically the role of preparatory steps prior to directional changes in 180° COD tasks.

Recent studies have used CMJ-RSImod as a measure of jump performance in various athletic populations [21,32,33]. However, to the author’s knowledge no investigation is yet to investigate its association with COD ability. In this investigation, CMJ-RSImod demonstrated significant moderate to large correlations with CODS. These results seem to be consistent with previous research [1,12,34] which found CMJ variables to relate to COD ability. It seems possible that the current results are due to CMJ-RSImod being an indicator of lower-body jump performance, supporting previous research which found measures of reactive strength to associate with COD ability [12]. Another important finding was that CMJ-RSImod demonstrated significant correlations with measures of COD deficit in pooled data and female players. The CMJ-RSImod calculates how high an athlete jumps in relation to how quickly the jump was performed, whereas the COD deficit is defined as how quickly an athlete can perform a COD in relation to their linear sprint speed. To exhibit a greater RSImod, an athlete must jump higher or decrease their time to take-off, or both. Similarly, if linear sprint speed remains constant, to improve COD deficit athletes must enter and exit the COD as quickly as possible. To do this, high amounts of force must be produced and maintained in both concentric and eccentric phases during CMJ [35,36] and COD [9,10,18,37]. Therefore, the ability to rapidly generate ECC and CON forces appear vastly important to RSImod and COD. When observing the correlations between CMJ-RSImod and measures of COD deficit, it is interesting to note no correlations were observed in male players. This finding is consistent with that of Lockie et al. [38] who found no associations between CMJ height and SLH to COD deficit in male soccer players. Nimphius et al. [29] suggest the COD deficit may provide an alternate measure of COD ability by removing the influence that linear speed may have on a COD test such as the 505 and 505_mod_. This may provide some reason as to why there were no significant associations between CMJ-RSImod and the COD deficit, as the influence of stretch-shortening capacities prevalent in the CMJ may not be present in the COD deficit in male athletes. These findings may help us to understand the determinants of both global and isolated measures of COD ability for both males and females, and how other factors such as technique may influence these relationships.

The results of this investigation indicate CMJ height to significantly relate to CODS. These results seem to be consistent with other research which found CMJ height to relate to COD in various populations [1,7,12,34,39,40]. These relationships may partly be explained by the fact that CMJ, 505, and 505_mod_ are dynamic movements requiring high levels of muscular power and, therefore should be closely related. Also, the correlation between CMJ height and COD ability appeared to be higher in female players, which agrees with previous research [39,41,42]. Another possible explanation for the association between CMJ height and COD ability is that CODS is highly influenced by linear sprint speed, which has been shown to correlate with CMJ height [1,34]. Furthermore, previous research demonstrates similar correlations between CMJ height and 180° COD tests [1,34]. It can thus be suggested that CMJ height is a strong determinant of COD ability. Therefore, when strength and power profiling athletes for monitoring and testing, researchers and practitioners are recommended to interpret CMJ height determined from vertical velocity at take-off as a determinant of CODS.

The current investigation found that SLH demonstrated significant moderate to large correlations with CODS. These results seem to be consistent with other research which found SLH to be associated with COD ability [14,16]. These results may be explained by the fact that the SLH is a measure of horizontal jump performance, which has been previously identified as an underpinning muscle strength quality of COD [43]. Another possible explanation for this is that the SLH consists of elements of force production for take-off and force acceptance when landing, which closely relates to the entry and braking demands of 180° COD ability [9,18,37]. These factors may also explain the reason why SLH testing a common practice for assessing both performance and injury risk factors in multiple populations [44,45,46]. A note of caution is due here since SLH did not seem to show as strong correlations to COD deficit as compared with CODS. It can therefore be assumed that the SLH is closely associated with both 505 and 505_mod_ CODS, but its association with the COD deficit remains unclear. Therefore, future studies which take these variables into account, will need to be undertaken.

Prior studies have noted the importance of maximal isometric strength for COD ability [7,40,47]. In this investigation, IMTP-PF demonstrated significant correlations with CODS in pooled and male data. These results are in line with those of previous research when reporting the importance of maximal isometric strength in male players [48,49]. This result may be explained by the fact that COD ability is a combination of all three strength abilities (CON, ECC, and isometric) [7]. Specifically, high levels of isometric strength appear to help maintain the required body position during the plant phase of the COD [7,8,10]. In contrast to earlier findings [7,8,40,41], no evidence of a significant relationship between IMTP-PF and COD ability was detected in female players. It is difficult to explain this result, yet it might be related to differences in sporting backgrounds between the studies or the statistical analyses performed in the current investigation. For example, this investigation performed correlational analyses on unilateral muscle strength qualities and matched COD limb, whereas previous research in female athletes performed analyses based on bilateral stance IMTP and COD testing retained the dominant limb for further analysis [7,8]. According to these findings, it can thus be suggested that maximal isometric strength does not play an important role in CODS in the current female cohort. Therefore, the present investigation raises the possibility that measures of jump performance (SLH, CMJ-RSImod and CMJ height) are stronger determinants of CODS in female athletes.

The current investigation found ECC-EXT to observe moderate associations with COD ability when tasks were analyzed on the right limb for male and pooled data. Similarly, pooled and male player analyses revealed significant moderate correlations between ECC-EXT with both 505 and 505_mod_ CODS for the left limb and when averaged across both limbs. However, ECC-EXT demonstrated no associations to CODS for female players for right and left limb, and when averaged across limbs. Furthermore, ECC-EXT showed no association with measures of COD deficit in any data set, or across limb analyses. Several reports have shown that ECC-EXT is largely associated with COD ability [1,9,18]. The associations in this investigation were lower compared to those of other studies. A possible explanation for this might be that this investigation matched variables according to limb, while previous research analyzed ECC-EXT of the dominant limb only [18]. It was expected that ECC-EXT would be associated with 505 and 505_mod_ to control knee flexion when GRFs are high during braking. This investigation has been unable to demonstrate any association between ECC-EXT and COD deficit measures. This result may be explained by the fact that COD deficit (the additional time taken to change direction compared to a linear sprint over an equivalent distance [29]) is highly influenced by isometric and reactive muscle strength qualities [7,8,10]. This notion may be supported by findings in the current investigation where isometric strength (IMTP-PF) and jump performances (SLH, CMJ-RSImod and CMJ height) demonstrated stronger associations to measures of COD deficit, as compared with ECC-EXT. It is therefore likely that such connections exist between such muscle strength qualities and COD deficit. It should be noted that correlations can only give insights into associations and not into causes and effects; therefore, the practical implications described previously need to be interpreted with this in mind.

Some limitations exist in the current study. Change of direction movements are multifactorial involving numerous variables to produce a faster performance [43]. Factors such as technique and muscle activation were not investigated and are limitations, as these factors also influence an athlete’s ability to produce a faster CODS. Although this investigation utilized a variety of court and field sport athletes, the results of this investigation are from a homogenous group with similar training backgrounds, playing experience, and current training schedules, making the findings less variable and thereby adding a degree of strength to the analysis.

## 6. Conclusions

The purpose of the current investigation was to explore the associations between unilateral muscle strength qualities and COD measures when matched limb-for-limb (i.e., right limb vs. right limb, left limb vs. left limb) in adolescent team-sport athletes. This investigation has shown that associations exist between unilateral muscle strength qualities and measures of COD ability. Coaches and strength and conditioning coaches should ensure that adolescent team-sport athletes develop multi-directional (horizontal and vertical) and unilateral muscle strength qualities, which should result in improvements in COD ability. Unilateral strength should be improved as part of a periodised training programme, ensuring that specific COD training is not neglected. It is acknowledged that future interventions should be undertaken to confirm or reject the causal relationships reported to fully understand the association between unilateral muscle strength qualities and COD ability.

## Figures and Tables

**Table 1:** Descriptive data for strength, power and COD measures across right, left, and both limbs averaged.

Variable	Pooled	Male	Female
	Mean ± SD	Mean ± SD	Mean ± SD
Right			
CMJ-RSImod (m·s^−1^)	0.18 ± 0.09	0.19 ± 0.05	0.16 ± 0.12
CMJ height (m)	0.13 ± 0.04	0.15 ± 0.04	0.11 ± 0.04
SLH (m)	1.47 ± 0.24	1.58 ± 0.24	1.37 ± 0.18
IMTP-PF (N·kg^−1^)	27.11 ± 5.31	28.82 ± 5.01	25.49 ± 5.11
ECC-EXT (Nm·kg^−1^)	3.31 ± 0.76	3.57 ± 0.86	3.06 ± 0.54
505 CODS (s)	2.47 ± 0.36	2.41 ± 0.15	2.60 ± 0.17
505_mod_ CODS (s)	2.77 ± 0.40	2.70 ± 0.15	2.93 ± 0.16
505 COD Deficit (s)	0.53 ± 0.15	0.52 ± 0.14	0.54 ± 0.14
505_mod_ COD Deficit (s)	0.83 ± 0.16	0.81 ± 0.13	0.87 ± 0.14
Left			
CMJ-RSImod (m·s^−1^)	0.17 ± 0.07	0.20 ± 0.06	0.15 ± 0.08
CMJ height (m)	0.13 ± 0.04	0.15 ± 0.04	0.11 ± 0.03
SLH (m)	1.48 ± 0.23	1.59 ± 0.23	1.37 ± 0.18
IMTP-PF (N·kg^−1^)	26.85 ± 5.14	28.37 ± 4.85	25.40 ± 5.03
ECC-EXT (Nm·kg^−1^)	3.17 ± 0.82	3.54 ± 0.82	2.81 ± 0.66
505 CODS (s)	2.49 ± 0.35	2.46 ± 0.14	2.61 ± 0.15
505_mod_ CODS (s)	2.78 ± 0.40	2.72 ± 0.16	2.92 ± 0.16
505 COD Deficit (s)	0.56 ± 0.12	0.57 ± 0.12	0.57 ± 0.10
505_mod_ COD Deficit (s)	0.84 ± 0.16	0.84 ± 0.13	0.87 ± 0.13
Average			
CMJ-RSImod (m·s^−1^)	0.17 ± 0.08	0.20 ± 0.05	0.16 ± 0.10
CMJ height (m)	0.13 ± 0.04	0.15 ± 0.04	0.11 ± 0.04
SLH (m)	1.47 ± 0.23	1.58 ± 0.22	1.37 ± 0.18
IMTP-PF (N·kg^−1^)	26.98 ± 5.16	28.60 ± 4.84	25.44 ± 5.02
ECC-EXT (Nm·kg^−1^)	3.24 ± 0.73	3.56 ± 0.79	2.94 ± 0.52
505 CODS (s)	2.52 ± 0.17	2.43 ± 0.13	2.60 ± 0.15
505_mod_ CODS (s)	2.82 ± 0.18	2.71 ± 0.15	2.92 ± 0.15
505 COD Deficit (s)	0.55 ± 0.11	0.55 ± 0.12	0.56 ± 0.11
505_mod_ COD Deficit (s)	0.84 ± 0.13	0.82 ± 0.12	0.87 ± 0.12

Notes: CMJ = countermovement jump; RSImod = reactive strength index-modified; SLH = single-leg hop; IMTP-PF = isometric mid-thigh pull peak force; ECC-EXT = eccentric extensor; 505_mod_ = modified 505; CODS = change of direction speed; COD deficit = change of direction deficit.

**Table 2:** Correlation coefficients between muscle strength qualities and COD measures on the right limb.

Variable	CODS		COD Deficit	
	505 R	505_mod_ R	505 R	505_mod_ R
Pooled (*n* = 115)				
CMJ-RSImod	−0.45 **	−0.50 **	−0.24 *	−0.34 **
CMJ height	−0.53 **	−0.60 **	−0.18	−0.30 *
SLH	−0.62 **	−0.64 **	−0.27 *	−0.32 *
IMTP-PF	−0.39 **	−0.44 **	−0.20	−0.40 **
ECC-EXT	−0.30 *	−0.45 **	−0.09	−0.20
Males (*n* = 56)				
CMJ-RSImod	−0.28	−0.32	−0.06	−0.22
CMJ height	−0.26	−0.38	−0.06	−0.27
SLH	−0.48 **	−0.53 **	−0.15	−0.23
IMTP-PF	−0.47 **	−0.51 **	−0.30	−0.39 *
ECC-EXT	−0.22	−0.34 *	−0.10	−0.26
Females (*n* = 59)				
CMJ-RSImod	−0.39 *	−0.39 *	−0.39 *	−0.35 *
CMJ height	−0.44 *	−0.38 *	−0.23	−0.13
SLH	−0.56 **	−0.48 **	−0.34 *	−0.22
IMTP-PF	−0.01	−0.03	−0.08	−0.27
ECC-EXT	−0.19	−0.31	−0.09	−0.05

Notes: CMJ = countermovement jump; RSImod = reactive strength index-modified; SLH = single-leg hop; IMTP-PF = isometric mid-thigh pull peak force; ECC-EXT = eccentric extensor; 505_mod_ = modified 505; CODS = change of direction speed; COD deficit = change of direction deficit; R = right. * Correlation significant at *p* ≤ 0.05; ** Correlation significant at *p* ≤ 0.001.

**Table 3:** Correlation coefficients between muscle strength qualities and COD measures on the left limb.

Variable	CODS		COD Deficit	
	505 L	505_mod_ L	505 L	505_mod_ L
Pooled (*n* = 115)				
CMJ-RSImod	−0.57 **	−0.57 **	−0.30 *	−0.36 **
CMJ height	−0.69 **	−0.65 **	−0.30 *	−0.34 **
SLH	−0.61 **	−0.65 **	−0.15	−0.31 *
IMTP-PF	−0.43 **	−0.40 **	−0.22	−0.26 *
ECC-EXT	−0.43 **	−0.44 **	0.05	−0.15
Males (*n* = 56)				
CMJ-RSImod	−0.39	−0.37	−0.33	−0.26
CMJ height	−0.54 **	−0.42 *	−0.45 **	−0.34
SLH	−0.55 **	−0.59 **	−0.19	−0.30
IMTP-PF	−0.57 **	−0.36	−0.44 *	−0.23
ECC-EXT	−0.38*	−0.39 *	−0.23	−0.27
Females (*n* = 59)				
CMJ-RSImod	−0.42 *	−0.44 **	−0.30	−0.45 **
CMJ height	−0.57 **	−0.51 **	−0.19	−0.31
SLH	−0.43 *	−0.49 **	−0.14	−0.28
IMTP-PF	−0.07	−0.12	0.01	−0.14
ECC-EXT	−0.13	−0.11	0.07	0.01

Notes: CMJ = countermovement jump; RSImod = reactive strength index-modified; SLH = single-leg hop; IMTP-PF = isometric mid-thigh pull peak force; ECC-EXT = eccentric extensor; 505_mod_ = modified 505; CODS = change of direction speed; COD deficit = change of direction deficit; L = left. * Correlation significant at *p* ≤ 0.05; ** Correlation significant at *p* ≤ 0.001.

**Table 4:** Correlation coefficients between muscle strength qualities and COD measures average across both limbs.

Variable	CODS		COD Deficit	
	505	505_mod_	505	505_mod_
Pooled (*n* = 115)				
CMJ-RSImod	−0.56 **	−0.57 **	−0.31 *	−0.38 **
CMJ height	−0.67 **	−0.67 **	−0.24	−0.34 **
SLH	−0.65 **	−0.67 **	−0.21	−0.33 **
IMTP-PF	−0.43 **	−0.44 **	−0.23	−0.34 **
ECC-EXT	−0.40 **	−0.49 **	−0.06	−0.20
Males (*n* = 56)				
CMJ-RSImod	−0.40 *	−0.40 *	−0.19	−0.26
CMJ height	−0.43 *	−0.43 *	−0.22	−0.31
SLH	−0.57 **	−0.62 **	−0.18	−0.30
IMTP-PF	−0.57 **	−0.48 **	−0.40 *	−0.36 *
ECC-EXT	−0.39 *	−0.42 *	−0.16	−0.22
Females (*n* = 59)				
CMJ-RSImod	−0.45 **	−0.42 *	−0.44 *	−0.42 *
CMJ height	−0.60 **	−0.49 **	−0.25	−0.23
SLH	−0.55 **	−0.51 **	−0.32	−0.27
IMTP-PF	−0.06	−0.08	−0.01	−0.21
ECC-EXT	−0.18	−0.23	−0.03	−0.04

Notes: CMJ = countermovement jump; RSImod = reactive strength index-modified; SLH = single-leg hop; IMTP-PF = isometric mid-thigh pull peak force; ECC-EXT = eccentric extensor; 505_mod_ = modified 505; CODS = change of direction speed; COD deficit = change of direction deficit. * Correlation significant at *p* ≤ 0.05; ** Correlation significant at *p* ≤ 0.001.

## Supplementary Materials

The following are available online at http://www.mdpi.com/2075-4663/6/3/83/s1, Table S1: title, Table S1 Correlation coefficients between muscle strength qualities and COD ability on the right limb. Table S2 Correlation coefficients between muscle strength qualities and COD measures on the left limb. Table S3 Correlation coefficients between muscle strength qualities and COD measures average across both limbs.

## References

[1] Jones P., Bampouras T.M., Marrin K. (2009). An investigation into the physical determinants of change of direction speed. J. Sports Med. Phys. Fit..

[2] Bloomfield J., Polman R., O’Donoghue P. (2007). Turning movements performed during FA Premier League soccer matches. J. Sports Sci. Med..

[3] Robinson G., O’Donoghue P., Wooster B. (2011). Path changes in the movement of English Premier League soccer players. J. Sports Med. Phys. Fit..

[4] Ade J., Fitzpatrick J., Bradley P.S. (2016). High-intensity efforts in elite soccer matches and associated movement patterns, technical skills and tactical actions. Information for position-specific training drills. J. Sports Sci..

[5] Sweeting A.J. (2017). Discovering the Movement Sequences of Elite and Junior Elite Netball Athletes.

[6] Young W.B., Henry B., Dawson G. (2015). Agility and change-of-direction speed are independent skills: Implications for training for agility in invasion sports. Int. J. Sports Sci. Coach..

[7] Spiteri T., Nimphius S., Hart N.H., Specos C., Sheppard J.M., Newton R.U. (2014). Contribution of Strength Characteristics to Change of Direction and Agility Performance in Female Basketball Athletes. J. Strength Cond. Res..

[8] Spiteri T., Newton R.U., Binetti M., Hart N.H., Sheppard J.M., Nimphius S. (2015). Mechanical Determinants of Faster Change of Direction and Agility Performance in Female Basketball Athletes. J. Strength Cond. Res..

[9] Graham-Smith P., Atkinson L., Barlow R., Jones P. (2009). Braking Characteristics and Load Distribution in 180 Degree Turns.

[10] Spiteri T., Cochrane J.L., Hart N.H., Haff G.G., Nimphius S. (2013). Effect of strength on plant foot kinetics and kinematics during a change of direction task. Eur. J. Sport Sci..

[11] Conlon J., Haff G.G., Nimphius S., Tran T., Newton R.U. (2013). Vertical jump velocity as a determinant of speed and agility performance. J. Aust. Strength Cond..

[12] Castillo-Rodríguez A., Fernández-García J.C., Chinchilla-Minguet J.L., Carnero E.Á. (2012). Relationship between muscular strength and sprints with changes of direction. J. Strength Cond. Res..

[13] Young W.B., James R., Montgomery I. (2002). Is muscle power related to running speed with changes of direction?. J. Sports Med. Phys. Fit..

[14] Meylan C., McMaster T., Cronin J., Mohammad N.I., Rogers C. (2009). Single-leg lateral, horizontal, and vertical jump assessment: Reliability, interrelationships, and ability to predict sprint and change-of-direction performance. J. Strength Cond. Res..

[15] Yanci J., Los Arcos A., Mendiguchia J., Brughelli M. (2014). Relationships between sprinting, agility, one-and two-leg vertical and horizontal jump in soccer players. Kinesiol. Int. J. Fundam. Appl. Kinesiol..

[16] Lockie R.G., Callaghan S.J., Berry S.P., Cooke E.R.A., Jordan C.A., Luczo T.M., Jeffriess M.D. (2014). Relationship Between Unilateral Jumping Ability and Asymmetry on Multidirectional Speed in Team-Sport Athletes. J. Strength Cond. Res..

[17] Brughelli M., Cronin J., Levin G., Chaouachi A. (2008). Understanding change of direction ability in sport: A review of resistance training studies. Sports Med..

[18] Jones P.A., Thomas C., Dos’Santos T., McMahon J.J., Graham-Smith P. (2017). The Role of Eccentric Strength in 180° Turns in Female Soccer Players. Sports.

[19] Dos’Santos T., Thomas C., Jones P.A., Comfort P. (2017). Asymmetries in single and triple hop are not detrimental to change of direction speed. J. Trainol..

[20] Suchomel T.J., Sole C.J., Bailey C.A., Grazer J.L., Beckham G.K. (2015). A comparison of reactive strength index-modified between six US collegiate athletic teams. J. Strength Cond. Res..

[21] Suchomel T.J., Bailey C.A., Sole C.J., Grazer J.L., Beckham G.K. (2015). Using reactive strength index-modified as an explosive performance measurement tool in Division I athletes. J. Strength Cond. Res..

[22] Owen N.J., Watkins J., Kilduff L.P., Bevan H.R., Bennett M.A. (2014). Development of a criterion method to determine peak mechanical power output in a countermovement jump. J. Strength Cond. Res..

[23] Munro A.G., Herrington L.C. (2011). Between-session reliability of four hop tests and the agility *T*-test. J. Strength Cond. Res..

[24] Stone M.H., Sands W.A., Carlock J., Callan S.A.M., Dickie D.E.S., Daigle K., Cotton J., Smith S.L., Hartman M. (2004). The Importance of Isometric Maximum Strength and Peak Rate-of-Force Development in Sprint Cycling. J. Strength Cond. Res..

[25] Maffiuletti N.A., Aagaard P., Blazevich A.J., Folland J., Tillin N., Duchateau J. (2016). Rate of force development: Physiological and methodological considerations. Eur. J. Appl. Physiol..

[26] Dos’ Santos T., Jones P.A., Comfort P., Thomas C. (2017). Effect of Different Onset Thresholds on Isometric Midthigh Pull Force-Time Variables. J. Strength Cond. Res..

[27] Graham-Smith P., Jones P.A., Comfort P. (2013). Assessment of Knee Flexor and Extensor Muscle Balance. Int. J. Athl. Ther. Train..

[28] Reilly T. (2006). The Science of Soccer Training: A Scientific Approach to Developing Strength, Speed and Endurance.

[29] Nimphius S., Callaghan S.J., Sptieri T., Lockie R.G. (2016). Change of direction deficit: A more isolated measure of change of direction performance than total 505 time. J. Strength Cond. Res..

[30] Cohen J. (1988). Statistical Power Analysis for the Behavioral Sciencies.

[31] Curtin F., Schulz P. (1998). Multiple correlations and Bonferroni’s correction. Biol. Psychiatry.

[32] Beckham G.K., Suchomel T.J., Bailey C.A., Sole C.J., Grazer J.L. The relationship of the reactive strength index-modified and measures of force development in the isometric mid-thigh pull. Proceedings of the 32 International Conference of Biomechanics in Sports.

[33] McMahon J.J., Jones P.A., Suchomel T.J., Lake J., Comfort P. (2017). Influence of Reactive Strength Index Modified on Force-and Power-Time Curves. Int. J. Sports Physiol. Perform..

[34] Hori N., Newton R.U., Andrews W.A., Kawamori N., McGuigan M.R., Nosaka K. (2008). Does Performance of Hang Power Clean Differentiate Performance of Jumping, Sprinting, and Changing of Direction?. J. Strength Cond. Res..

[35] Cormie P., McGuigan M.R., Newton R.U. (2010). Adaptations in athletic performance after ballistic power versus strength training. Med. Sci. Sports Exerc..

[36] Cormie P., McGuigan M.R., Newton R.U. (2010). Changes in the eccentric phase contribute to improved stretch-shorten cycle performance after training. Med. Sci. Sports Exerc..

[37] Dos’ Santos T., Thomas C., Jones P.A., Comfort P. (2017). Mechanical determinants of faster change of direction speed performance in male athletes. J. Strength Cond. Res..

[38] Lockie R.G., Stage A.A., Stokes J.J., Orjalo A.J., Davis D.L., Giuliano D.V., Moreno M.R., Risso F.G., Lazar A., Birmingham-Babauta S.A. (2016). Relationships and predictive capabilities of jump assessments to soccer-specific field test performance in Division I collegiate players. Sports.

[39] Barnes J.L., Schilling B.K., Falvo M.J., Weiss L.W., Creasy A.K., Fry A.C. (2007). Relationship of jumping and agility performance in female volleyball athletes. J. Strength Cond. Res..

[40] Thomas C., Comfort P., Jones P.A., Dos’Santos T. (2017). A Comparison of Isometric Mid-Thigh Pull Strength, Vertical Jump, Sprint Speed, and Change of Direction Speed in Academy Netball Players. Int. J. Sports Physiol. Perform..

[41] Nimphius S., McGuigan M.R., Newton R.U. (2010). Relationship between Strength, Power, Speed, and Change of Direction Performance of Female Softball Players. J. Strength Cond. Res..

[42] Vescovi J.D., McGuigan M.R. (2008). Relationships between sprinting, agility, and jump ability in female athletes. J. Sports Sci..

[43] Sheppard J.M., Young W.B. (2006). Agility literature review: Classifications, training and testing. J. Sports Sci..

[44] Bossuyt F.M., Garcia-Pinillos F., Raja Azidin R.M., Vanrenterghem J., Robinson M.A. (2016). The Utility of a High-intensity Exercise Protocol to Prospectively Assess ACL Injury Risk. Int. J. Sports Med..

[45] Myers B.A., Jenkins W.L., Killian C., Rundquist P. (2014). Normative data for hop tests in high school and collegiate basketball and soccer players. Int. J. Sports Phys. Ther..

[46] Noyes F.R., Barber S.D., Mangine R.E. (1991). Abnormal lower limb symmetry determined by function hop tests after anterior cruciate ligament rupture. Am. J. Sports Med..

[47] Dos’Santos T., Thomas C., Jones P.A., Comfort P. (2018). Asymmetries in Isometric Force-Time Charcteristics Are Not Detrimental to Change of Direction Speed. J. Strength Cond. Res..

[48] Thomas C., Dos’Santos T., Comfort P., Jones P.A. (2016). Relationship between Isometric Strength, Sprint, and Change of Direction Speed in Male Academy Cricketers. J. Trainol..

[49] Thomas C., Comfort P., Chiang C., Jones P.A. (2015). Relationship between isometric mid-thigh pull variables and sprint and change of direction performance in collegiate athletes. J. Trainol..

