# Supplementary material for: Relationships between Unilateral Muscle Strength Qualities and Change of Direction in Adolescent Team-Sport Athletes
Source: Sports (Basel). 2018 Aug 20;6(3):83. doi: 10.3390/sports6030083 (PMC6162690; doi:10.3390/sports6030083)
Supplement: Supplementary file 1 [file sports-06-00083-s001.pdf]

Relationships between Unilateral Muscle Strength Qualities and Change of Direction in Adolescent Team-Sport Athletes

Table S1. Correlation coefficients between muscle strength qualities and COD ability on the right limb.

Variable	CODS		COD Deficit	
	**505 R**	**505_{mod} R**	**505 R**	**505_{mod} R**
Soccer				
CMJ-RSImod	−0.54*	−0.66**	−0.16	−0.38
CMJ height	−0.55*	−0.68**	−0.08	−0.29
SLH	−0.70**	−0.77**	−0.27	−0.38
IMTP-PF	−0.14	−0.18	0.09	−0.03
ECC-EXT	−0.39	−0.56*	−0.21	−0.38
Cricket				
CMJ-RSImod	−0.53**	−0.53**	−0.20	−0.25
CMJ height	−0.58**	−0.58**	−0.20	−0.26
SLH	−0.64**	−0.71**	−0.32	−0.43*
IMTP-PF	−0.66**	−0.70**	−0.37*	−0.50**
ECC-EXT	−0.39	−0.47*	−0.20	−0.30
Court				
CMJ-RSImod	−0.18	−0.23	−0.23	−0.27
CMJ height	−0.45*	−0.49*	−0.10	−0.19
SLH	−0.60**	−0.47*	−0.30	−0.35
IMTP-PF	−0.26	−0.20	0.08	−0.16
ECC-EXT	−0.02	−0.31	−0.11	0.01

Notes: CMJ = countermovement jump; RSImod = reactive strength index-modified; SLH = single-leg hop; IMTP-PF = isometric mid-thigh pull peak force; ECC-EXT = eccentric extensor; 505_{mod} = modified 505; CODS = change of direction speed; COD deficit = change of direction deficit; L= left.*Correlation significant at $p \leq 0.05$. **Correlation significant at $p \leq 0.001$.

Table S2. Correlation coefficients between muscle strength qualities and COD measures on the left limb.

Variable	CODS		COD Deficit	
	**505 L**	**505_{mod} L**	**505 L**	**505_{mod} L**
Soccer				
CMJ-RSImod	−0.71**	−0.65**	−0.41	−0.27
CMJ height	−0.66**	−0.61**	−0.38	−0.27
SLH	−0.71**	−0.71**	−0.22	−0.26
IMTP-PF	0.07	0.11	0.11	0.14
ECC-EXT	−0.32	−0.42	−0.05	−0.25
Cricket				
CMJ-RSImod	−0.58**	−0.55**	−0.16	−0.22
CMJ height	−0.72**	−0.71**	−0.23	−0.28
SLH	−0.62**	−0.70**	−0.15	−0.43*
IMTP-PF	−0.71**	−0.66**	−0.39*	−0.37*

ECC-EXT	−0.56**	−0.50**	−0.22	−0.26
Court				
CMJ-RSImod	−0.27	−0.45*	−0.32	−0.44*
CMJ height	−0.68**	−0.64**	−0.34	−0.38
SLH	−0.47*	−0.54**	−0.32	−0.37
IMTP-PF	−0.47*	−0.33	−0.15	−0.20
ECC-EXT	−0.35	−0.36	−0.01	−0.05

Notes: CMJ = countermovement jump; RSImod = reactive strength index-modified; SLH = single-leg hop; IMTP-PF = isometric mid-thigh pull peak force; ECC-EXT = eccentric extensor; 505_{mod} = modified 505; CODS = change of direction speed; COD deficit = change of direction deficit; L= left. *Correlation significant at $p \leq 0.05$. **Correlation significant at $p \leq 0.001$.

Table S3. Correlation coefficients between muscle strength qualities and COD measures average across both limbs.

Variable	CODS		COD Deficit	
	**505**	**505_{mod}**	**505**	**505_{mod}**
Soccer				
CMJ-RSImod	−0.66**	−0.69**	−0.31	−0.35
CMJ height	−0.59**	−0.62**	−0.31	−0.32
SLH	−0.73**	−0.76**	−0.33	−0.35
IMTP-PF	−0.02	−0.03	0.16	0.15
ECC-EXT	−0.36	−0.53*	−0.13	−0.41
Cricket				
CMJ-RSImod	−0.59**	−0.55**	−0.27	−0.29
CMJ height	−0.75**	−0.70**	−0.32	−0.33
SLH	−0.67**	−0.73**	−0.33	−0.47*
IMTP-PF	−0.73**	−0.70**	−0.42*	−0.48*
ECC-EXT	−0.54**	−0.55**	−0.20	−0.26
Court				
CMJ-RSImod	−0.28	−0.35	−0.27	−0.36
CMJ height	−0.53*	−0.59**	−0.19	−0.31
SLH	−0.59**	−0.57**	−0.32	−0.40*
IMTP-PF	−0.40	−0.30	−0.04	−0.22
ECC-EXT	−0.17	−0.36	0.13	−0.02

Notes: CMJ = countermovement jump; RSImod = reactive strength index-modified; SLH = single-leg hop; IMTP-PF = isometric mid-thigh pull peak force; ECC-EXT = eccentric extensor; 505_{mod} = modified 505; CODS = change of direction speed; COD deficit = change of direction deficit. *Correlation significant at $p \leq 0.05$. **Correlation significant at $p \leq 0.001$.
